# F-18-FDG and C-11-Choline Positron Emission Tomography in Human Esophago-Gastric Cancer: Prediction of Response to Therapy

**DOI:** 10.4021/wjon2010.04.201w

**Published:** 2010-04-30

**Authors:** Stuart Suttie, Dympna McAteer, Margaret Sheehan, Marianne Nicolson, Lutz Schweiger, Solveig Hammonds, Timothy Smith, Andrew Welch, Kenneth Park

**Affiliations:** a Department of Surgery, Aberdeen Royal Infirmary, Foresterhill, Aberdeen, UK, AB25 2ZN; b Department of Radiology, Aberdeen Royal Infirmary, Foresterhill, Aberdeen, UK, AB25 2ZN; c Department of Pathology, Aberdeen Royal Infirmary, Foresterhill, Aberdeen, UK, AB25 2ZN; d Department of Oncology, Aberdeen Royal Infirmary, Foresterhill, Aberdeen, UK, AB25 2ZN; e Department of Biomedical Physics, Aberdeen University, Aberdeen Royal Infirmary, Foresterhill, Aberdeen, UK, AB25 2ZN

**Keywords:** Positron emission tomography, Esophageal cancer, F-18-FDG, C-11-Choline, Response

## Abstract

**Background:**

To determine the utility of F-18-FDG and C-11-Choline uptake, in patients with esophageal and esophago-gastric junction tumors who are to undergo either neo-adjuvant or palliative chemotherapy, in predicting response (pathological and survival).

**Methods:**

Eighteen patients with biopsy proven cancer were recruited prospectively. Patients underwent PET imaging before and during the first cycle of chemotherapy (seven and 14 days) with both F-18-FDG and C-11-Choline. Tracer uptake was quantified using Standardized Uptake Values. Pathological tumor response was determined using the Mandard criteria. Cellular proliferation was determined using ki-67 immunohistochemistry. Relationships between tracer uptake and response, one-year survival and cellular proliferation were determined.

**Results:**

All 18 tumors were imaged by F-18-FDG PET compared to 16/18 with C-11-Choline. Change in uptake of either tracer did not correlate with pathological response. Pathological response did not influence survival (median-survival, responders = 16.1 months; non-responders = 19.0 months, *p* = 0.978). There was no significant correlation of change in tracer uptake with survival. C-11-Choline tumor uptake did not correlate with cellular proliferation.

**Conclusion:**

F-18-FDG PET is superior for imaging of the primary tumor. Neither F-18-FDG nor C-11-Choline PET was able to predict response accurately.

## Introduction

Imaging modalities utilized in identifying response to therapy in gastro-esophageal cancer, include computerized tomography (CT) and endoscopic ultrasound (EUS), detecting anatomical changes within the tumor. These changes take several months to become apparent and may be indistinguishable from surrounding tissue edema. Positron Emission Tomography (PET) utilizes metabolically active tracers to probe metabolic changes associated with response that precede morphological changes [1, 2]. Esophageal cancer has a poor prognosis [3], worse in those having no response to neo-adjuvant chemotherapy than patients proceeding directly to surgery. PET has potential benefits, theoretically allowing decisions regarding adjustments in chemotherapy to be made during the course of treatment.

[^18^F]2-fluoro-2-deoxy-d-glucose (F-18-FDG) is the most commonly used tracer for PET imaging and has been successful at imaging [4] and identifying response to therapy [5-7] in gastro-esophageal malignancies.

Carbon-11-Choline (C-11-Choline) PET has not been used to monitor response to chemotherapy in patients with gastro-esophageal cancers to date. Choline is a precursor for the synthesis of phospholipids and is required for transmembrane signalling and lipid-cholesterol transport [8, 9]. Carcinogenesis and tumor cell growth are characterized by increased cellular proliferation and increased cell membrane synthesis. C-11-Choline uptake is thought to parallel cell proliferation, hence its theoretical use in predicting response.

The primary aim of our study was to determine if C-11-Choline was superior to F-18-FDG PET for predicting pathological response and survival, in patients with esophageal and esophago-gastric junction tumors undergoing either neo-adjuvant or palliative chemotherapy. The secondary aim was to determine if C-11-Choline uptake correlated with cellular proliferation.

## Patients and Methods

### Patients

The Grampian Research Ethics Committee approved the study. Eighteen patients with biopsy proven esophageal/esophago-gastric junction (OGJ) cancers undergoing neo-adjuvant or palliative chemotherapy were prospectively recruited. Before chemotherapy, all patients underwent staging CT. A subset of patients underwent EUS as part of the COGNATE trial [10]. Restaging following initial therapy was performed at the discretion of the primary surgeon/oncologist, based on initial staging and clinical response to chemotherapy.

Patient inclusion criteria: 1) Biopsy proven esophageal cancer (adenocarcinoma/squamous cell carcinoma); 2) Biopsy proven OGJ cancer (Siewert type I + II) [11]; 3) Eligible to receive neo-adjuvant/palliative chemotherapy [12, 13].

Patient exclusion criteria: 1) Diabetes mellitus

Patients who had histo-pathology and stage discussed at the weekly Multi-Disciplinary-Team-meeting, and judged suitable for curative surgery, underwent neoadjuvant chemotherapy prior to their surgery. Early in the study this incorporated cisplatin and 5-fluorouracil as per OE02 trial [12]. Latterly 5-fluorouracil was substituted with capecitabine (1250 mg/m^2^ daily).

A subset of patients who had unresectable locally advanced disease at staging, but with adequate response to therapy, may become candidates for surgery had three cycles of triple combination therapy (epirubicin, cisplatin and capecitabine [13]) prior to restaging with CT. In patients with significant cardiac disease, epirubicin was substituted with mitomycin-C (60 mg/m^2^, alternate cycles). Upon restaging, if disease progression was identified and the tumor deemed unresectable the patient underwent a course of concurrent chemoradiotherapy over five weeks (radiotherapy: 50 Gy, 25 daily fractions for five weeks; chemotherapy: 33 days of capecitabine in 1300 mg/m^2^/day with cisplatin in 20 mg/m^2^ weekly for five weeks).

Patients with advanced disease entered into the REAL-2 study [13]. Patients with significant cardiac disease had epirubicin substituted with mitomycin-C (off-trial), and received three cycles then re-staged with CT. If there was a radiological response a further three cycles were given.

Baseline PET scanning was performed in the two week interval prior to commencing chemotherapy, then at day seven and 14 from the onset of first cycle of chemotherapy, using both F-18-FDG and C-11-Choline.

### PET protocol

PET scans were performed on a CTI/Siemens ECAT-EXACT-31-scanner (Knoxville, Tennessee, USA). Patients were fasted for 6 hours prior to scanning to reduce circulating glucose levels and enhance F-18-FDG uptake. Blood glucose levels were measured prior to scanning. Patients Body Surface Area (BSA) were calculated according to the formula: BSA (m^2^) = 0.20247 x Height (cm)^0.725^xWeight (kg)^0.425^ [14] . Tumor site was marked on the anterior chest wall using information from CT and endoscopy, allowing a 10 cm window to be identified around the tumor. Subsequent PET scans were based on this one bed, in 2D acquisition mode, with transmission scans utilising a Gadolinium source. Images were reconstructed using filtered back projection with a Hanning window (cut-off at the Nyquist-frequency) and calibrated by imaging a cylindrical phantom of F-18-FDG on the same day as each image. F-18-FDG and C-11-Choline were manufactured on site. C-11-Choline studies were performed prior to the F-18-FDG due to Carbon-11s shorter half life.

### C-11-Choline

A 10-minute transmission scan was acquired for attenuation correction over the 10cm bed previously identified. All patients received an intravenous bolus of C-11-Choline (375 MBq; mean 318 MBq, range 198 - 396 MBq) after which imaging was immediately commenced for 40 minutes for a dynamic acquisition scan (2 x 15 second frames, 3 x 30 second frames, 3 x 60 second frames, 5 x 180 second frames and 4 x 300 second frames).

Patients were then allowed to move around freely for 40 minutes before commencing the F-18-FDG study.

### F-18-FDG

A 10-minute transmission scan was acquired for attenuation correction over the 10 cm bed previously identified. An intravenous bolus of F-18-FDG (185 MBq; mean 177 MBq, range 106 - 238 MBq) was used to keep the cumulative effective dose low (in line with European recommendations for injected F-18-FDG activity [15]). Imaging was immediately commenced for 60 minutes for a dynamic acquisition scan (8 x 15 second frames, 4 x 30 second frames, 1 x 60 second frame, 1 x 300 second frame and 5 x 600 second frames).

### Quantification

SUV_BSA_ analysis was performed on all PET scans. A static image containing the data for the last 10 minutes of the dynamic acquisition was used to define the boundaries of the visible tumor. A standardised uptake value, scaled by body surface area (SUV_BSA_), was obtained from this final frame of data.

Regions of Interest (ROI) were drawn around the visible tumor in multiple slices obtained from the static image using a tumor threshold of 42% of the maximum-pixel-value within the tumor. The maximum SUV_BSA_ was utilized.

### Imaging

Staging CT and EUS were performed within two weeks of baseline PET. Staging was performed as per criteria laid out by the American Joint Committee on Cancer [16], and blinded to pathological staging.

### Surgery

Surgery was performed three to four weeks following completion of neo-adjuvant chemotherapy.

### Pathology

Surgical specimens were fixed in formaldehyde and assessed for pathological stage [16] and Tumor Regression Grade (TRG), using the Mandard criteria [17]. A pathological response was defined as a TRG of 1-3, whilst a TRG score of 4-5 indicated little or no response. The resection was assessed for completeness of resection (R_0_, complete tumor resection; R_1_, positive microscopic tumor margin).

### Ki-67 immunohistochemical analysis

Ki-67 immunohistochemical analysis was performed on formalin fixed, paraffin embedded tumor tissue taken at the time of diagnostic endoscopy and surgical resection specimen. Paraffin embedded sections 3 - 4 µm thick were taken from each tumor sample and de-waxed and rehydrated prior to antigen retrieval. Each section was labelled using the monoclonal mouse antibody Ki-67, according to manufacturers’ protocol (clone MIB-1, DakoCytomation, Cambridgeshire, UK) at 1:500 dilution following antigen retrieval by microwaving the sections (20 minutes in microwave at 800 watts) in citrate buffer (0.01M, pH 6, in house solution). Antibody to antigen binding was detected using the avidin-biotin complex method (as per Dako protocol, DakoCytomation, Cambridgeshire, UK). Immunohistochemistry was performed on an automated stainer (TechMate, DakoCytomation, Cambridgeshire, UK). Each run included positive and negative controls. For positive controls sections of human reactive lymph node were used with a high mitotic/proliferative rate. Primary antibodies were omitted on sections used as negative controls.

The number of ki-67 positive tumor nuclei were counted in two to five (dependent on amount of tissue available) high power fields (x40) for each sample of tumor with the proliferative activity expressed as the mean number of positively stained tumor nuclei in all fields assessed (± standard deviation) as per Breeuwsma et al [18]. All of the fields examined represented tumor with no normal tissue within those fields. The pathologist was blinded to the PET results.

### Survival

All patients were followed up for a minimum of one year or until death.

### Statistics

Spearman Rank correlation was performed to assess the relationship between pathological response, ki-67 proliferative activity and initial tumor tracer uptake as well as percentage change in tumor uptake of each PET tracer at seven and 14 days into the first cycle of neoadjuvant chemotherapy.

Influence of pathological response, resection status, initial and percentage change in PET tracer uptake on survival was assessed using Kaplan Meier survival curves (log rank statistic).

Analysis was performed on the Statistical Package for Social Sciences (Statistical Package for the Social Sciences V13.1, Chicago, USA). A *p*-value of less than 0.05 denotes significance.

## Results

Eighteen patients, 12 male and six female, with a mean age of 60.9 years (range 50.1 - 78.1) were recruited. The majority of cancers were adenocarcinomas (16/18, 89%; 2/18, 11% were squamous cell) and were esophageal in origin (10/18, 56%; 8/18, 44% were OGJ in origin). Most patients presented with stage III and IV disease (12/18, 67%), Table 1. The mean blood glucose, prior to each PET scan was 5.6 mmol/l (range 4.4 - 7.7 mmol/l).

**Table 1:** Patients, tumour characteristics, staging and chemotherapy regimens

Patient	Sex	Age	Tumour Location	Tumour Histology	Pre Treatment AJCC Stage	Staging Modality	Chemotherapy	Chemotherapy Regime	Chemotherapy Cycles Completed	Re staging (CT)	Further Treatment
1	M	55.5	E	A	III	CT	MCX	Palliative	3	-	-
2	M	54.4	EGJ	A	IIA	CT	CF	Neoadjuvant	2	IIA	Surgery
3	F	67.6	E	A	III	CT	CF	Neoadjuvant	2	-	Surgery
4	M	50.9	EGJ	A	IIA	CT	CF	Neoadjuvant	2	IIA	Surgery
5	M	50.1	E	S	IIA	CT/EUS	CF	Neoadjuvant	2	-	Surgery
6	F	69.4	E	A	IIA	CT	CX	Neoadjuvant	2	IIA	CRT
7	M	56.5	EGJ	A	III	CT/EUS	CX	Neoadjuvant	2	III	CRT
8	M	61.9	E	A	IVB	CT	ECF	Palliative	0	-	-
9	M	78.1	EGJ	A	IVB	CT	ECF	Palliative	4	IVB	ECF
10	F	70.7	E	A	III	CT	CX	Neoadjuvant	2	-	Surgery
11	M	52.5	EGJ	A	IVA	CT	CX	Neoadjuvant	2	-	Surgery
12	M	54.3	E	A	IIA	CT/EUS	CX	Neoadjuvant	2	IIA	Surgery
13	M	63.3	EGJ	A	IIA	CT	ECX	Neoadjuvant	3	III	CRT
14	F	60.0	EGJ	A	IVB	CT	MCX	Palliative	3	IVB	MCX
15	F	67.9	E	S	IVA	CT	MCX	Neoadjuvant	3	III	Surgery
16	F	64.7	E	A	III	CT	CX	Neoadjuvant	2	III	Surgery
17	M	57.9	E	A	III	CT	CX	Neoadjuvant	2	IVA	CRT
18	M	59.7	EGJ	A	III	CT	ECX	Neoadjuvant	3	IVA	CRT

(M = male; F = female; E = esophagus; EGJ = esophago-gastric junction; A = adenocarcinoma; S = squamous cell carcinoma; CT = computerised tomography; EUS = endoscopic ultrasound; E = epirubicin; C = cisplatin; F =5-fluorouracil; X = capecitabine; M = mitomycin-C; CRT = chemoradiotherapy)

Of 18 patients recruited into this study, 14 initially underwent neoadjuvant chemotherapy with only nine of these patients proceeding to surgery (Table 2). Two patients, upon completion of neoadjuvant chemotherapy refused surgery and underwent chemoradiotherapy instead (patients six and seven). Three patients had progression of disease during neoadjuvant chemotherapy and after discussion at the multi-disciplinary-team-meeting, surgery was substituted with chemoradiotherapy (patients 13, 17 and 18). Of four patients receiving palliative chemotherapy, only two completed the full course (patients nine and 14). Of the remaining two, one had clinical progression of disease (patient one) and underwent stenting after completing only three cycles of chemotherapy whilst the second patient died from an aorto-esophageal fistula prior to chemotherapy (patient eight).

**Table 2:** Survival, Pathological Response, Resection Status and Ki-67 Status

Patient	Chemotherapy Regime	TRG	Resection Status	Pathological Stage	Survival (months)	Status at Follow Up	Median Ki-67 Tumour Nucleii in pre-treatment biopsy (± SD)	High Power Fields Counted	Mean ki-67 Tumour Nuclei in Resection Specimen (± SD)	High Power Fields Counted	% Δ in Ki-67 Staining
1	Palliative				6.0	Dead	236 (55)	5			
2	Neoadjuvant	4	R_1_	T3 N1 Mx	7.6	Dead					
3	Neoadjuvant	3	R_1_	T2 N1 Mx	8.4	Dead	117 (48)	5	85 (44)	6	-27.4
4	Neoadjuvant	3	R_0_	T3 N1 Mx	15.8	Dead	228 (72)	4	203 (41)	5	-11.0
5	Neoadjuvant	5	R_0_	T3 N0 Mx	25.1	Alive	276 (39)	5	184 (46)	6	-33.3
6	Neoadjuvant				24.4	Alive	395 (43)	5			
7	Neoadjuvant				17.9	Dead	186 (43)	5			
8	Palliative				0.3	Dead	741 (79)	3			
9	Palliative				9.2	Dead	466 (51)	5			
10	Neoadjuvant	4	R_1_	T3 N1 Mx	11.9	Dead	575 (63)	4	135 (24)	15	-76.5
11	Neoadjuvant	2	R_0_	T3 N1 Mx	20.0	Alive	616 (31)	4	73 (35)	7	-88.1
12	Neoadjuvant	4	R_1_	T3 N1 Mx	19.8	Alive	194 (82)	5	149 (30)	7	-23.2
13	Neoadjuvant				18.2	Alive	437 (107)	5			
14	Palliative				10.0	Dead	35 (5)	2			
15	Neoadjuvant	2	R_0_	T2 N0 Mx	16.4	Alive	209 (42)	5	0 (0)	5	-100.0
16	Neoadjuvant	4	R_0_	T3 N1 Mx	15.4	Alive					
17	Neoadjuvant				13.8	Alive	536 (76)	4			
18	Neoadjuvant				13.7	Alive	437 (158)	5			

### PET imaging

All 18 patients underwent baseline PET imaging with both F-18-FDG and C-11-Choline. Two patients underwent no further PET imaging following baseline PET due to one patient (patient six) suffering toxic side effects of chemotherapy and therefore unable to travel for PET imaging and one patient (patient eight) who died prior to chemotherapy. At day 14, patient 12 underwent imaging with F-18-FDG alone (problems with C-11-Choline synthesis). F-18-FDG PET detected more extensive local disease than seen on staging CT and endoscopy in patient 18, which was confirmed on re-staging CT scan. There was no C-11-Choline tumor uptake prior to chemotherapy in patients 11 and 18.

All 18 patients (100%) had their primary tumor imaged with F-18-FDG in contrast to just 16 (89%) primary tumors successfully imaged with C-11-Choline. The two primary tumors not successfully imaged with C-11-Choline (patient 11 and 18) were sited at the esophago-gastric junction, with a high uptake of C-11-Choline within the liver obscuring the primary tumor. Neither C-11-Choline nor F-18-FDG could differentiate between primary tumors and involved peri-esophageal lymph nodes.

### Tumor uptake

There was no correlation between initial F-18-FDG and C-11-Choline uptake (r = -0.07, *p* = 0.80) with no significant difference between mean initial uptake for F-18-FDG (0.19) and C-11-Choline (0.18), *p* = 0.88.

There was no significant correlation of percentage change in tracer uptake at day seven (F-18-FDG vs C-11-Choline, r = -0.33, *p* = 0.33) and at day 14 (r = -0.33, *p* = 0.38).

### Pathological response

**Figure 1:**
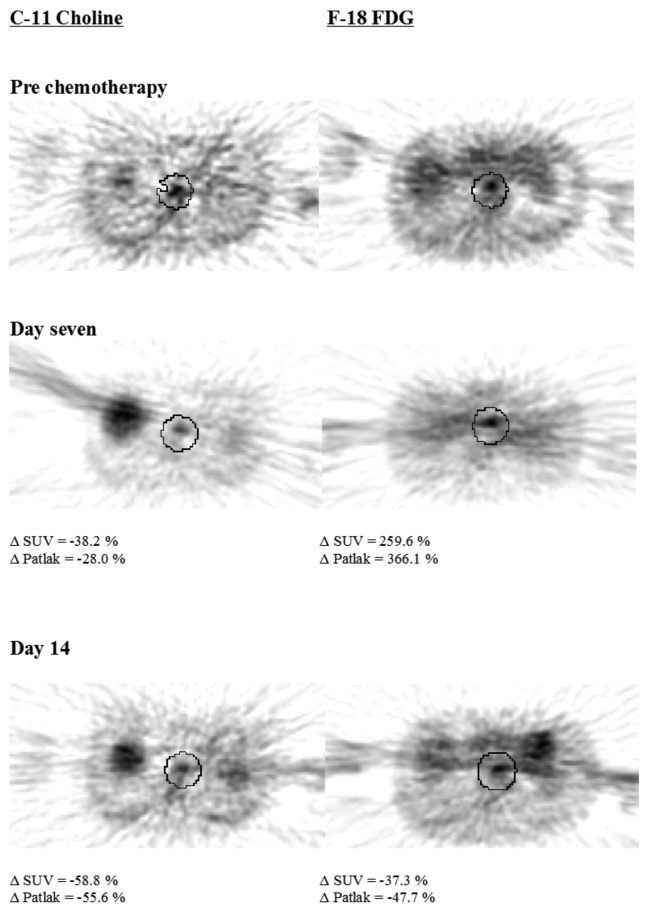
Trans-axial PET images of patient three, a pathological responder.

Nine of 14 patients initially receiving neoadjuvant chemotherapy proceeded to surgery. Four of these nine (44%) had a pathological response (TRG 2-3), with five patients undergoing a curative (R_0_) resection (Table 2). Figure 1 displays images obtained with each PET tracer.

There was no significant correlation of the initial tumor tracer uptake for either F-18-FDG (n = 9, r = -0.17, *p* = 0.66) or C-11-Choline (n = 8, r = 0.51, *p* = 0.19) with pathological response. There was no significant correlation between percentage change in uptake of either tracer and pathological response or TRG at day seven or 14, although percentage change in C-11-Choline uptake at day 14 and TRG showed a trend towards significance (r = 0.95, *p* = 0.05).

### Pathological response, resection status and survival

**Figure 2:**
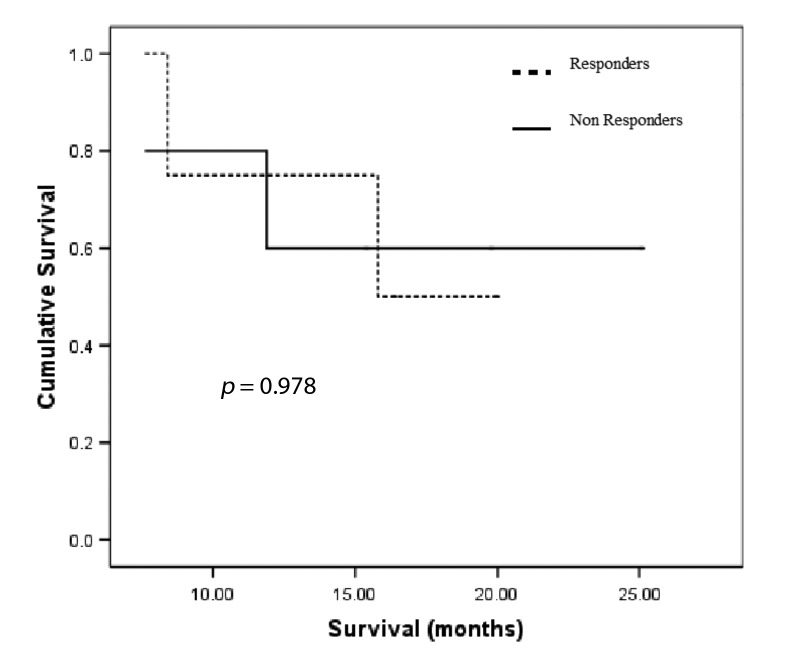
Kaplan Meier survival curves for pathological response.

Follow-up ranged from 0.3 - 25.1 months. There was no significant difference in median survival between pathological responders (n = 4, 16.1 months) and non-responders (n = 5, 19.0 months), *p* = 0.98 (Fig. 2). Resection status had no significant impact on survival (R_0_: n = 5, 22.8 months; R_1_: n = 4, 11.9 months), *p* = 0.07.

For survival analysis the patients were dichotomised based on the median initial tumor PET tracer uptake (i.e. greater or less than the median tumor PET tracer uptake) or on median percentage change in uptake (i.e. greater or less than the median % Δ tumor PET tracer uptake). There was no significant difference in survival between the two groups (Table 3a, b).

**Table 3a:** Median Initial Tumor PET Tracer Uptake and Survival

Quantification	Tracer	Median Uptake (± SD)	Survival, months (± SD)	p	n
SUV	F-18 FDG	0.17 (0.06)	< median 14.8 (2.0) > median 17.2 (2.7	0.70	18
	C-11 Choline	0.15 (0.01)	< median 19.3 (2.7) > median 11.5 (2.3)	0.11	16

**Table 3b:** Median % Δ in SUV PET Tumor Tracer Uptake at Seven and 14 days into the First Cycle of Chemotherapy and Survival

PET Timing	Tracer	Median % Δ SUV (± SD)	Survival, months (± SD)	p	n
Day 7	F-18 FDG	-3.3 (81.9)	< Median 15.0 (2.3) > Median 20.7 (2.7)	0.43	13
	C-11 Choline	0.0 (24.1)	< Median 21.3 (3.4) > Median 14.1 (2.1)	0.24	11
Day 14	F-18 FDG	-17.1 (28.3)	< Median 18.1 (1.2) > Median 17.2 (3.3)	0.41	12
	C-11 Choline	-0.2 (36.9)	< Median 21.1 (3.4) > Median 13.2 (2.1)	0.16	9

### Ki-67 staining

Ki-67 immunohistochemical staining was performed in 16 out 18 patients on the pre treatment endoscopic biopsy (Table 2). All pre treatment tumor tissue showed positive ki-67 nuclear staining. All patients receiving neo-adjuvant chemotherapy had a reduction ki-67 nuclear staining (Table 2).

There was no significant correlation of the mean number of ki-67 positive tumor nuclei in pre treatment tumor samples with initial tumor uptake of either F-18-FDG or C-11-Choline, (C-11-Choline: r = -0.17, *p* = 0.57, n = 14; F-18-FDG: r = 0.24, *p* = 0.37, n = 16). There was no significant correlation of change in ki-67 tumor staining with change in tumor tracer uptake over time (F-18-FDG day 7: r = 0.31, *p* = 0.54, n = 6; day14: r = -0.40, *p* = 0.51, n=5: C-11-Choline day 7: r = -0.60, *p* = 0.29, n = 5; day14: r = -0.50, *p* = 0.67, n = 3).

There was no significant correlation of the pre-treatment tumor ki-67 staining with pathological response (r = -0.14, *p* = 0.76, n = 7) and percentage change in tumor ki-67 staining and pathological response (r = -0.14, *p* = 0.76, n = 7).

For survival analysis the patients were dichotomised based on the median number of tumor nuclei staining positive for ki-67 in pre treatment samples. The median number of ki-67 positive tumor nuclei was 336 per high power field per sample. There was no significant difference in survival (more than median ki-67 staining: 17.9 months ± -3.1, n = 8; less than median: 15.8 months ± 2.6, n = 8), *p* = 0.51.

The median change in ki-67 expression following neoadjuvant chemotherapy was -33.3% (SD ± 35.8). For survival analysis, the patients were divided into two groups based on median change in ki-67 histochemical expression. There was no significant difference in the median survival between those patients with a greater than median reduction in ki-67 staining (14.7 months ± 2.7, n = 5) and those with a lesser reduction in ki-67 expression (21.8 months ± 2.9, n = 3), *p* = 0.32.

## Discussion

The majority of studies utilizing PET to monitor response to treatment in esophago-gastric cancers have employed F-18-FDG with no studies using C-11-Choline. C-11-Choline has only been used in two studies for imaging of esophago-gastric cancers [19, 20].

### Imaging of tumor

In this study F-18-FDG successfully imaged all 18 primary tumors, irrespective of tumor location and histological type. C-11-Choline was unable to image two primaries, both substantial adenocarcinomas at the OGJ. This limitation of C-11-Choline PET is related to the considerable non specific uptake of C-11-Choline within the liver [19, 20]. A further hypothesis for this was proposed by Jager et al [19], stating that primary tumors had a considerable lower SUV uptake of C-11-Choline thereby causing difficulty in distinguishing tumor from normal tissue. This finding may be due to differing methods of quantification, with our study showing no such difference in uptake. Kobori et al [20] found C-11-Choline to be more sensitive in identifying small tumors than F-18-FDG, whilst Jager et al found that irrespective of tumor size, F-18-FDG was superior at imaging of the primary tumor. In our study, neither tracer was able to distinguish primary tumor from peri-esophageal disease, in keeping with previous reports [19].

### Pathological response

A pathological response was achieved in 44% of patients in this study, consistent with population based data [21]. This study found no significant correlation of pre-treatment tumor uptake of C-11-Choline or F-18-FDG with pathological response, in keeping with previous reports [22, 23]. We found, as did others [22, 24], that change in uptake of either tracer at each time point could not significantly distinguish between each TRG. The percentage change in C-11-Choline at day 14 strongly correlated with TRG (r = 0.95, *p* = 0.05), although there were few responders in this group. When TRG are amalgamated into groups indicating either responders or non responders, the change in C-11-Choline uptake appears to have some success in distinguishing between them. Only five studies have assessed F-18-FDG and pathological response during the course of chemotherapy [6] or chemoradiotherapy [2, 23, 25, 26]. Wieder et al performed F-18-FDG PET after two weeks and upon completion of chemoradiotherapy for squamous cell carcinoma of the esophagus [23]. Using ROC analysis, a percentage change in SUV uptake of 30% (at two weeks) was able to distinguish responders from non responders. Kroep et al, using F-18-FDG PET after the second of six cycles of chemotherapy for both adenocarcinoma and squamous cell carcinoma, reported a similar finding, although they identified the threshold change in SUV for predicting response at 40% [6]. Using F-18-FDG PET to predict pathological response earlier than two weeks into the treatment regime appears to be too early in order to distinguish responders from non responders [25]. A recent study looking at change in F-18-FDG uptake during and upon completion of therapy found no correlation between tracer uptake and pathological response [26]. Studies identifying a link between change in F-18-FDG uptake and pathological response, did so with a high sensitivity and low specificity (range 55-86 %) for identifying responders [6, 23, 27] (i.e. up to 45% of non-responders would be classified as responders according to F-18-FDG PET). Studies have shown that patients with a poor response to neoadjuvant therapy have a worse prognosis than those who did not have neoadjuvant therapy [28]. It is these non responders whom we need to identify, accurately and at an early stage so that treatment can be altered.

### Survival

We found that pathological response did not predict one year survival, although the analysis only included nine patients. Wieder and Brucher et al found that change in F-18-FDG correlated with pathological response, which in turn conferred a survival advantage [23, 27]. In a study designed to evaluate the impact of pathological response, Dunne et al reported that pathological response was not an independent prognostic indicator [29], a finding reiterated by others [30]. A limitation of assessing pathological response, is that subjective criteria, open to sampling errors are used. We found that at day 14 into the first cycle of chemotherapy, patients with a greater reduction in C-11-Choline, had a worse prognosis than those with a smaller reduction. This phenomenon may relate to tumors with a high proliferation rate are more responsive to therapy [31, 32]. The only factors strongly influencing survival in our study were the presence of a curative resection and the change in C-11-Choline uptake at day 14.

### Cellular proliferation

All pre-treatment tumors were positive for ki-67, with all who received neoadjuvant chemotherapy having a reduction in ki-67 staining. Choline and its metabolites have been found in increased levels in a variety of tumors [33, 34]. Cell populations with a high proliferative activity are known to have higher rates of choline transport into cells compared with slower growing populations [35, 36] suggesting a correlation between choline uptake and proliferation [37-42]. Several studies reported that F-18-FDG uptake correlates with viable cell number rather than proliferation [43-46], although recent studies have reported a correlation between ki-67 staining and F-18-FDG uptake in lung, brain and ovarian tumors [47-50], but not in esophageal cancers [45]. This study found no correlation of ki-67 staining with initial C-11-Choline or F-18-FDG tumor uptake. This absence of correlation with pre-therapy tumors may be due to the heterogeneous nature of tumors even within a single tumor type. In some cases where we obtained multiple samples from single tumors we observed a wide variation in ki-67 protein expression. In contrast the PET tracer incorporation was determined on the whole tumor. Two studies assessing correlation of C-11-Choline uptake with ki-67 proliferative index in brain and prostate tumors came to opposite conclusions [18, 40]. Breeuwsma et al found that C-11-Choline uptake within prostate cancer showed no significant correlation with ki-67 [18], whilst Utriainen et al reported a strong correlation of ki-67 proliferative index with C-11-Choline uptake [40]. Using proton magnetic resonance spectroscopy they found that the concentration of choline containing compounds within the tumors investigated correlated with the ki-67 proliferation index although there was no correlation of the concentration of the choline compounds with C-11-Choline uptake [40].

### Limitations

In keeping with the majority of PET studies, the major limitation was patient numbers. Only recently has an adequately powered F-18-FDG PET study been performed, utilizing F-18-FDG to predict response to neoadjuvant chemotherapy as well as influence treatment [51] in OGJ cancers. The patients were a heterogeneous group, in terms of chemotherapy (which may have an effect on response as well as tumor tracer uptake [52]), tumor types and stage, all of which may have had an impact.

F-18-FDG PET is superior for imaging of esophageal and OGJ cancers. Neither tracer was able to predict pathological response or survival. C-11-Choline uptake did not directly relate to cellular proliferation.
